# From the heart to the mind: cardiac vagal tone modulates top-down and bottom-up visual perception and attention to emotional stimuli

**DOI:** 10.3389/fpsyg.2014.00278

**Published:** 2014-05-01

**Authors:** Gewnhi Park, Julian F. Thayer

**Affiliations:** ^1^Department of Psychology, Azusa Pacific UniversityAzusa, CA, USA; ^2^Department of Psychology, The Ohio State UniversityColumbus, OH, USA

**Keywords:** cardiac vagal tone, emotion regulation, spatial frequency, perception, attention

## Abstract

The neurovisceral integration model (Thayer and Lane, 2000) posits that cardiac vagal tone, indexed by heart rate variability (HRV), can indicate the functional integrity of the neural networks implicated in emotion–cognition interactions. Our recent findings begin to disentangle how HRV is associated with both top-down and bottom-up cognitive processing of emotional stimuli. Higher resting HRV is associated with more adaptive and functional top-down and bottom-up cognitive modulation of emotional stimuli, which may facilitate effective emotion regulation. Conversely, lower resting HRV is associated with hyper-vigilant and maladaptive cognitive responses to emotional stimuli, which may impede emotion regulation. In the present paper, we recapitulate the neurovisceral integration model and review recent findings that shed light on the relationship between HRV and top-down and bottom-up visual perception and attention to emotional stimuli, which may play an important role in emotion regulation. Further implications of HRV on individual well-being and mental health are discussed.

When the mind is strongly excited, we might expect that it would instantly affect in a direct manner the heart; and this is universally acknowledged and felt to be the case. Claude Bernard also repeatedly insists, and this deserves especial notice, that when the heart is affected it reacts on the brain; and the state of the brain again reacts through the pneumo-gastric nerve on the heart; so that under any excitement there will be much mutual action and reaction between these, the two most important organs of the body.

Darwin, 1872, p. 69

As far back as the early 20th century, the work of Claude Bernard highlighted the importance of the brain–heart connection in understanding the interplay between emotion and cognition. More recently, Thayer and Lane (2000, 2002) proposed the neurovisceral integration model, which suggests that neural networks implicated in autonomic, emotional, and cognitive self-regulation are also involved in the control of cardiac autonomic activity. Behavioral and neuroimaging studies have identified several pathways by which cardiac vagal tone is linked to neural networks implicated in emotional and cognitive self-regulation (for a review, see Thayer et al., 2009). Our recent research begins to uncover how cardiac vagal tone indexed by heart rate variability (HRV) is associated with top-down and bottom-up visual perception and attention to emotional stimuli, which may play a critical role in regulating the impact of negative emotion – termed as *emotion regulation* (Gross, 1998; Gross and Thompson, 2007). Higher resting HRV is associated with more adaptive top-down and bottom-up cognitive modulation of emotional stimuli, which may allow for effective regulation of the impact of negative emotion (Gross, 1998; Gross and Thompson, 2007). In contrast, lower resting HRV is associated with hyper-vigilant and maladaptive cognitive responses to emotional stimuli, which may be detrimental to emotion regulation. The present paper briefly describes the neurovisceral integration model and then reviews our recent experiments that shed light on the interaction between cardiac vagal tone and top-down and bottom-up visual perception and attention to emotional stimuli.

## SELF-REGULATION AND THE CENTRAL AUTONOMIC NETWORK (CAN)

**FIGURE 1:**
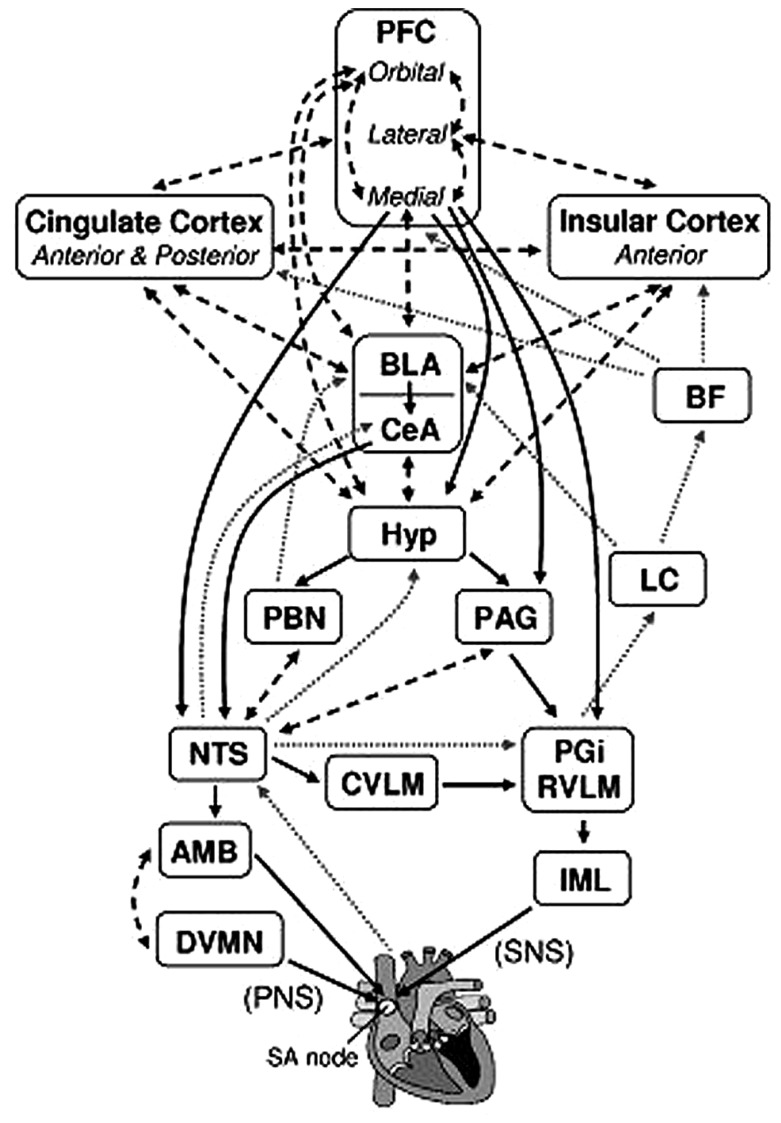
**Brain structures associated with the control of heart rate.** Solid black arrows indicate efferent pathways to the heart, including right vagus nerve (PNS) and stellate ganglion (SNS) inputs to the SA node. Dotted gray arrows indicate afferent pathways to medullary structures via aortic baroreceptor signals carried through the vagus. Dashed black arrows indicate bidirectional connections. AMB, nucleus ambiguus; BF, basal forebrain; BLA, baso-lateral amygdala; CeA, central nucleus of the amygdala; CVLM, caudal ventrolateral medullary neurons; DVMN, dorsal vagal motor nuclei; Hyp, hypothalamus (lateral and paraventricular); IML, intermediolateral cell column of the spinal cord; LC, locus coeruleus; NTS, nucleus of the solitary tract; PAG, periaqueductal gray; PBN, parabrachial nucluei; PFC, prefrontal cortex; PGi, nucleus paragigantocellularis; RVLM, rostral ventrolateral medullary neurons. We adapted and modified the diagram from Ellis and Thayer (2010; with permission of authors).

Self-regulation refers to the ability to regulate thoughts, emotions, and behaviors, thereby allowing people to choose responses that are appropriate for different situational demands (Gross, 1998; Thayer and Lane, 2000; Segerstrom and Nes, 2007; Park et al., 2014). Several neural mechanisms associated with cognitive, emotional, and autonomic self-regulation have been identified, one of which is the *central autonomic network* (CAN; Benarroch, 1993; Thayer and Lane, 2000; Thayer et al., 2009). The CAN has been implicated in making visceromotor, neuroendocrine, and behavioral responses that are adaptive and flexible for various environmental demands (Thayer and Lane, 2000; Thayer et al., 2009; Park et al., 2013a). The structures of the CAN include the anterior cingulate, the insula, the ventromedial prefrontal cortices, the central nucleus of the amygdala, the paraventricular and related nuclei of the hypothalamus, the periaquaductual gray matter, the parabrachial nucleus, the nucleus of the solitary tract (NTS), the nucleus ambiguous, the ventrolateral medulla, the ventromedial medulla, and the medullary tegmental field, among others (see Figure 1; Thayer et al., 2009; Ellis and Thayer, 2010). These brain structures in the CAN are reciprocally connected, and information can flow in both top-down and bottom-up fashions (Thayer and Lane, 2000). They are also loosely connected so that it is easy to recruit additional structures that are necessary to make specific behavioral changes (Thayer and Lane, 2000).

In particular, the prefrontal-subcortical inhibitory circuits within the CAN play a critical role in self-regulatory function (for a review, see Heatherton and Wagner, 2011). Under normal circumstances, the prefrontal cortex identifies safety cues from the environment and exerts its inhibitory control over sympathoexcitatory subcortical circuits, including the central nucleus of the amygdala (Thayer et al., 2009; Heatherton and Wagner, 2011). This prefrontal regulation makes it possible for an organism to make responses that are optimal for different situational demands (Thayer et al., 2009; Park et al., 2013a). In threatening and uncertain situations, the prefrontal inhibitory regulation diminishes and sympathoexcitatory subcortical circuits make default threat responses (Park et al., 2013a). As such, prefrontal-subcortical inhibitory circuits play a role in various regulatory behaviors, including appetite, attitudes, and prejudice (Heatherton and Wagner, 2011). Reduced prefrontal regulation can lead to hyperactive subcortical activity, which results in prolonged activation of defensive behavior mechanisms, including hyper-vigilance and perseverative cognition (e.g., worry or rumination; Thayer et al., 2009; Park et al., 2013a). Not surprisingly, the disruption of prefrontal-subcortical circuits has been associated with a wide range of psychopathologies, including depression (Davidson et al., 2002; Johnstone et al., 2007), anxiety (Kim and Whalen, 2009), schizophrenia (Callicott et al., 2003; Lewis et al., 2005), and addictive behavior (for a review, see Li and Sinha, 2008).

## THE NEUROVISCERAL INTEGRATION MODEL AND HEART RATE VARIABILITY

According to the neurovisceral integration model (Thayer and Lane, 2000; Thayer et al., 2009), the functioning of prefrontal-subcortical inhibitory circuits critical for self-regulation is linked with the heart via the vagus nerve that provides inhibitory inputs to the heart (see also Levy, 1971; Benarroch, 1993; Ellis and Thayer, 2010). Several neuroimaging and pharmacological studies have identified the link between inhibitory prefrontal-subcortical circuits and cardiac vagal tone indexed by vagally mediated resting HRV (Task Force of the European Society of Cardiology, and the North American Society of Pacing, and Electrophysiology, 1996; Ahern et al., 2001; Lane et al., 2009; for a review, see Thayer et al., 2009). Most noticeably, a recent meta-analysis (Thayer et al., 2012) revealed that resting HRV is tied to the functioning of prefrontal-subcortical circuits, such that higher resting HRV is associated with the effective functioning of prefrontal-subcortical inhibitory circuits that support flexible and adaptive responses to environmental demands (Thayer and Lane, 2000; Thayer et al., 2009). Indeed, research has indicated that people with higher resting HRV exhibit effective behavioral responses (e.g., faster response times and better accuracy) on executive cognitive tasks (Hansen et al., 2003) as well as flexible and adaptive emotional responding (Ruiz-Padial et al., 2003; Thayer et al., 2009). In contrast, lower resting HRV is associated with hypoactive prefrontal regulation; this results in hyperactive subcortical structures, which leads to maladaptive cognitive and emotional self-regulation. For example, people with lower resting HRV often fail to recognize safety cues or to habituate to novel, neutral stimuli (hyper-vigilance; for a review, see Hansen et al., 2003; Friedman, 2007; Park et al., 2012a). Additionally, people with lower resting HRV responded to neutral stimuli with heightened startle and neural responses as if the stimuli were emotionally negative (Ruiz-Padial et al., 2003; Park et al., 2012a). Evidently there is a link between HRV and the prefrontal-subcortical circuits critical for cognitive and emotional self-regulation.

## HRV AND TOP-DOWN AND BOTTOM-UP VISUAL PERCEPTION OF EMOTIONAL STIMULI

We investigated whether resting HRV is associated with top-down and bottom-up visual perception of emotional facial expressions. To further isolate neuro-cognitive mechanisms, we presented faces at different spatial frequencies which are defined as the energy distribution in the scale specified as the number of cycles per degree of visual angle and/or the number of cycles per image (see Figure 2 from Parker et al., 1996; Morrison and Schyns, 2001; Park et al., 2013b). Broad spatial frequency (BSF) images contain all spatial frequency ranges and can be filtered to contain either high or low spatial frequencies (Vuilleumier et al., 2003; Holmes et al., 2005; Park et al., 2012a,b). Research has indicated that distinctive visual pathways are selectively sensitive to different ranges of spatial frequency information (Goffaux et al., 2005). For example, the *parvocellular* pathway, which mediates perception of color and contrast, is sensitive to high spatial frequency (HSF) information (Merigan and Maunsell, 1993; Vuilleumier et al., 2003). HSF fearful faces, in particular, elicited greater activity in the cortical structures, including the posterior cingulate, the motor cortex, the medial prefrontal cortex, and the lateral orbitofrontal cortex (Vuilleumier et al., 2003;Winston et al., 2003). The *magnocellular* pathway, which mediates perception of depth, motion, and low contrast black-and-white information, is sensitive to low spatial frequency (LSF) information (Merigan and Maunsell, 1993; Livingstone and Hubel, 1988; Vuilleumier et al., 2003; Nieuwenhuis et al., 2008; Park et al., 2012a,b). LSF fearful faces, in particular, are processed via the *retinotectal* pathway, the phylogenetically old visual pathway that directs information from the retina through the superior colliculus and pulvinar nucleus of the thalamus to the amygdala (Merigan and Maunsell, 1993; Livingstone and Hubel, 1988; Vuilleumier et al., 2003; Nieuwenhuis et al., 2008). As a result, greater amygdala activity was elicited by blurred and coarse LSF fearful faces (Vuilleumier et al., 2003). Therefore, emotional messages at LSF appear to be processed directly via the amygdala and subcortical mechanisms (Park et al., 2012b).

**FIGURE 2:**
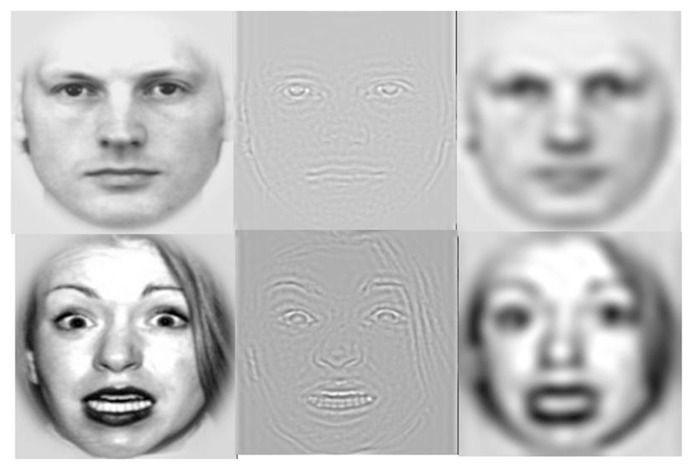
**Example stimuli from Park et al. (2012b).** Normal broad spatial frequency (BSF) fearful and neutral faces (left column), high spatial frequency (HSF) faces (middle column), and low spatial frequency (LSF) faces (right column).

It has been suggested that discriminating emotions using HSF information is difficult, whereas discriminating emotions using LSF information is relatively easy (Mermillod et al., 2008). When statistical properties of fearful and neutral faces with different spatial frequency information were analyzed, the statistical distributions of HSF fearful and neutral faces overlapped completely so that it was difficult for observers to discriminate emotion using HSF information; however, the statistical distributions of LSF fearful and neutral faces were so distinctive that it was easy to discriminate emotion using LSF information (Mermillod et al., 2008). As such, discriminating emotions using LSF is an easy task, whereas discriminating emotions using HSF is a difficult task that may require greater executive function mediated by the prefrontal cortex. In our recent study (Park et al., 2012b), participants were presented with fearful and neutral faces at BSF, HSF, and LSF for 200 ms and asked to discriminate the emotion of each face as quickly and accurately as possible. The results showed that people with higher resting HRV were capable of identifying HSF fearful faces more accurately than people with lower resting HRV. Thus, as predicted, there was a positive association between HRV and task performance (e.g., accuracy) on discriminating the emotion of HSF fearful faces.

Furthermore, we examined whether the top-down influence of different processing goals would modulate the relationship between HRV and the bottom-up visual perception of HSF fearful faces. A previous study revealed that the utilization of spatial frequency information depends on processing goals (Schyns and Oliva, 1999). When people were asked to discriminate the *emotion* of hybrid face stimuli (i.e., happy, neutral, and angry), they primarily utilized LSF information. In contrast, when people were asked to discriminate whether the stimuli were *expressive* or *not*, they utilized HSF information. Thus, the utilization of HSF information facilitates the discrimination of expressiveness (Schyns and Oliva, 1999). Consistent with this view, our results showed that the positive relationship between resting HRV and task performance observed in the emotion discrimination task diminished in the expressiveness discrimination task. The studies provided initial evidence that cardiac vagal tone is positively associated with the bottom-up processing of visual discrimination of emotion in HSF fearful faces, which is modulated by the top-down influence of different processing goals.

## HRV AND TOP-DOWN AND BOTTOM-UP EMOTIONAL ATTENTION

Research has indicated that neural structures implicated in attentional systems, such as the pulvinar, the cingulate, and the fronto-parietal cortex, influence and are influenced by affective processing (Okon-Singer et al., 2013; for a review, see Pessoa and Adolphs, 2011). Also, attentional systems play an important role in emotional regulation by allowing an organism to select and focus on optimal responses from a broad behavioral repertoire and to inhibit less functional responses (Gross, 1998; Thayer and Lane, 2000; Park et al., 2013b). Our research demonstrates that individual differences in resting HRV are associated with the ability to control different attentional systems in response to emotional stimuli.

**FIGURE 3:**
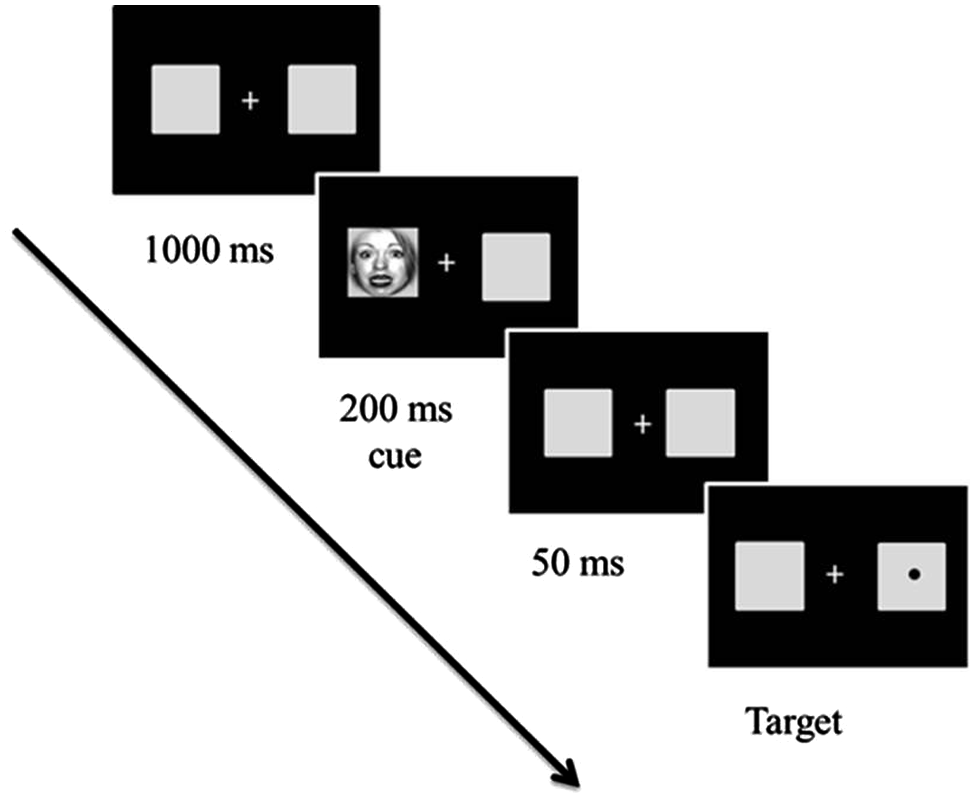
**Sample trial in Experiment 1 from Park et al. (2013b).** The cues and targets were equally likely to appear on the right or left of fixation. However, 80% of trials were valid (96 trials) and 20% of the trials were invalid (24 trials). The initial fixation display appeared for 1000 ms. Then, cues, which were either fearful or neutral faces created at broad, high, and low spatial frequency, appeared for 250 ms. After a 50 ms delay with the initial fixation display, a target circle appeared in the center of the left or right box until the participant responded (or until 2,000 ms elapsed). Stimuli are not drawn to scale.

*Emotional attention* refers to a phenomenon in which emotional stimuli are more likely to modulate one’s attention (Pourtois and Vuilleumier, 2006; see Vuilleumier and Brosch, 2009, for a review). Emotional attention has been extensively studied using Posner’s spatial cuing paradigm (for a review, see Posner and Peterson, 1990; Posner and Rothbart, 2007) to disentangle how different attentional components are involved in affective processing (Vuilleumier, 2005; Pourtois and Vuilleumier, 2006). In the spatial cuing task (Posner and Peterson, 1990; Bartolomeo et al., 2001; Posner and Rothbart, 2007), a cue can either accurately predict the location of a subsequent target (*valid*) or not (*invalid*; Posner and Peterson, 1990; Posner and Rothbart, 2007; see Figure 3). Two different types of attentional orienting have been identified: (a) *exogenous* and (b) *endogenous* attentional orienting (Posner and Peterson, 1990; Bartolomeo et al., 2001; Posner and Rothbart, 2007). Exogenous orienting is characterized as a bottom-up, reflexive orienting mode that is associated with neural activity of the posterior attention system, which includes the superior parietal cortex, pulvinar, and superior colliculus (Posner and Peterson, 1990; Huang-Pollock and Jigg, 2003; Berger et al., 2005; Posner and Rothbart, 2007). Exogenous orienting is typically observed when the duration between the onset of a cue and the onset of a target [stimulus-onset asynchrony (SOA)] is short, peaking at 150 ms. Endogenous orienting is characterized as a top-down, voluntary attentional mode, and is associated with the anterior attention system, which includes the anterior cingulate and prefrontal cortex (Posner and Peterson, 1990; Huang-Pollock and Jigg, 2003; Berger et al., 2005; Posner and Rothbart, 2007). Endogenous orienting is typically observed when the majority of the cues are valid at long SOAs (e.g., >300 ms). People notice that a target is more likely to appear where a preceding cue is presented and strategically exert voluntary attentional control (Coull et al., 2000; Bartolomeo et al., 2001).

A number of empirical studies have reported that when emotional stimuli (e.g., fearful faces) are presented as cues in the task, people are faster to detect targets in valid trials (faster attentional engagement) and slower to disengage attention away from cues in invalid trials (slower attentional disengagement; Mathews et al., 1997; Fox et al., 2002; Koster et al., 2006; Verkuil et al., 2009; Cisler and Koster, 2010). However, faster engagement to and slower disengagement from fearful faces are construed as maladaptive because faster engagement indicates hyper-vigilance to threatening stimuli, and slower attentional disengagement indicates the failure to inhibit attention from them (Fox et al., 2002; Verkuil et al., 2009). For example, it has been well established that people with high anxiety exhibit faster attentional engagement to and slower attentional disengagement from threatening stimuli compared to healthy controls (Mathews et al., 1997; Koster et al., 2006; Cisler and Koster, 2010).

Neuroimaging studies have revealed that fearful face cues facilitate attentional engagement through the neural mechanisms of the posterior attentional system, which includes the superior parietal cortex, pulvinar, and superior colliculus (Pourtois et al., 2005). Therefore, the neural mechanisms associated with exogenous orienting are also involved in controlling attentional engagement to fearful faces cues. Not only that, but the same subcortical structures are implicated in processing LSF fearful faces. Taking the evidence together, attentional engagement to LSF fearful face cues under the exogenous attention mode can be construed as a bottom-up mode of emotional attention because they are all associated with activity in subcortical neural mechanisms (Park et al., 2013b).

In contrast, attentional disengagement from fearful face cues in invalid trials is associated with increased activity of the ventromedial prefrontal cortex, including the rostral anterior cingulate cortex (Pourtois and Vuilleumier, 2006). Thus, both attentional disengagement from fearful face cues and endogenous attention are associated with activity in prefrontal neural structures. Not only that, but the prefrontal structures are also implicated in processing HSF fearful faces. Taking the evidence together, attentional disengagement from HSF fearful face cues under the endogenous mode can be construed as a top-down mode of emotional attention because they are all associated with activity in cortical neural mechanisms (Park et al., 2013b). We then examined the relationship between HRV and top-down and bottom-up aspects of emotional attention (Park et al., 2013b).

People with lower resting HRV showed significantly faster attentional engagement to LSF fearful faces relative to people with higher resting HRV at short SOAs (250 ms; Experiment 1), suggesting hyper-vigilant responses to threatening stimuli primilarly tapping into subcortical mechanisms. However, with longer SOAs, (960 ms), people with lower resting HRV showed significantly slower attentional disengagement from HSF fearful faces relative to people with higher resting HRV, suggesting the failure to inhibit attention from threatening stimuli primarily tapping into cortical mechanisms. These findings provide initial evidence that individual differences in resting HRV are associated with top-down and bottom-up emotional attention. People with higher resting HRV – associated with highly functional emotional and cognitive self-regulatory systems – show more adaptive top-down and bottom-up emotional attention, which may facilitate effective emotion regulation. In contrast, people with lower resting HRV – associated with poor emotional and cognitive self-regulatory systems – show maladaptive top-down and bottom-up emotional attention, which may be detrimental to emotion regulation.

In addition, another study by our group provides further evidence that individual differences in resting HRV predict the functioning of inhibitory attentional mechanisms critical for top-down emotional attention (Park et al., 2012c). The inhibition of return (IOR) refers to the inhibitory attentional mechanism that prevents one’s attention from going back to previously attended areas, thereby facilitating adaptive search (Posner and Cohen, 1984; Posner et al., 1985; Klein and MacInnes, 1999; Taylor and Therrien, 2005; Sumner, 2006; Stoyanova et al., 2007). Although people’s attention is initially drawn to the location of a stimulus, it is diverted to explore new locations when the location of the stimuli is irrelevant to the task and there is time to shift (Park et al., 2012c). Extensive research has revealed that there are at least two separate neural mechanisms of IOR: (a) *collicular* (*retinotectal*) and (b) *cortical* mechanisms, including the temporo-parietal cortex and anterior cingulate cortex, which are typically associated with attentional control. We examined whether individual differences in HRV would predict the IOR to emotional and neutral face cues (Park et al., 2012c). Furthermore, we explored whether the relationship between HRV and IOR to emotional stimuli depended on different neural pathways of IOR.

To dissociate the roles of the collicular and cortical pathways of IOR, we utilized LSF and HSF fearful facial stimuli, which are selectively sensitive to collicular (retinotectal) and cortical mechanisms, respectively (Park et al., 2012c). In general, people with lower resting HRV failed to demonstrate the IOR effect, indicating reduced inhibitory function. In contrast, people with higher resting HRV demonstrated a typical IOR effect, which was even more pronounced in response to HSF fearful faces that tapped into the cortical based IOR (Sumner, 2006). These findings demonstrate that people with higher resting HRV are capable of controlling inhibitory attention, highly instrumental in top-down emotional attention, whereas people with low resting HRV are less capable of controlling it.

## PHASIC HRV AND COGNITIVE EMOTION REGULATION

There is a growing body of evidence suggesting that changes in cardiac activity indexed by phasic HRV are linked with self-regulatory effort (Butler et al., 2006; Segerstrom and Nes, 2007; Gaebler et al., 2013; Park et al., 2014). However, whether phasic HRV *suppression* or *enhancement* is associated with the exertion of self-regulatory effort may depend on the context in which phasic HRV changes occur (Park et al., 2014). Phasic HRV suppression has been construed as an autonomic response to stress, which reflects the withdrawal of cardiac vagal control and the activation of the defensive systems to cope with a stressor (Thayer et al., 1996; Park et al., 2014). For example, when people were exposed to a video clip triggering stress such as depicting an escalating conflict (Beauchaine et al., 2007; El-Sheikh et al., 2011), asked to perform a difficult mental stress task (Weber et al., 2010), or engaging in worry and fear or anger imagery (Lyonfields et al., 1995; Thayer et al., 1996), phasic HRV suppression was observed. Conversely, phasic HRV enhancement has been construed as the exertion of self-regulatory effort while engaging in emotional regulation (e.g., employing emotion self-regulatory methods such as suppression or reappraisal) or performing a task that requires self-regulatory effort (Butler et al., 2006; Segerstrom and Nes, 2007). For example, phasic HRV enhancement was observed when participants were instructed to eat only carrots, which demanded greater self-regulatory effort, compared to when instructed to eat only cookies, demanded less self-regulatory effort. Phasic HRV enhancement was even more pronounced in people with higher resting HRV (Segerstrom and Nes, 2007). Additionally, greater phasic HRV enhancement was observed, when female participants were exposed to an upsetting film and then asked to regulate emotion by engaging in emotion suppression (i.e., suppressing emotional expressions) or emotion reappraisal (i.e., reappraising a situation to generate desired emotions), compared to controls (Butler et al., 2006). Thus, engaging in both emotion suppression and reappraisal was associated with phasic HRV enhancement. Furthermore, a recent neuroimaging finding revealed that phasic HRV enhancement was correlated with greater activation in the subgenual anterior cingulate cortex associated with emotional regulation (Lane et al., 2013).

**FIGURE 4:**
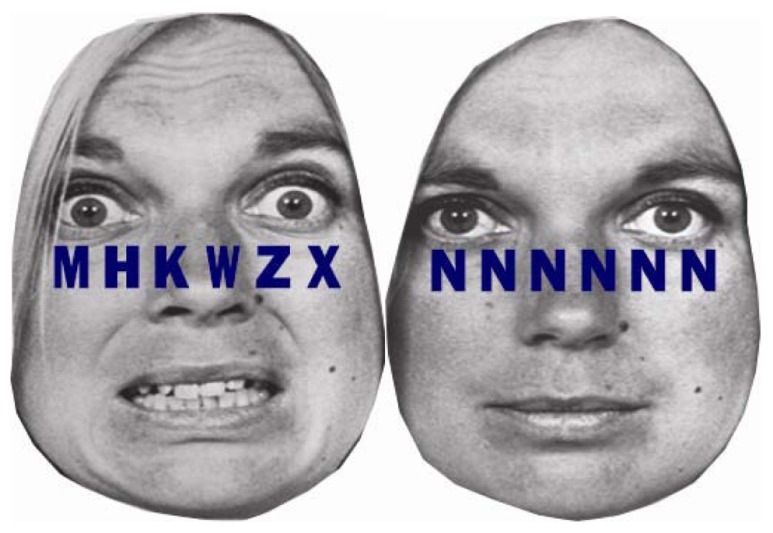
**Example stimuli from Park et al. (2013a, 2014).** A string of six letters was superimposed on fearful and neutral facial emotions. In the high cognitive load condition, letter strings consisted of one target letter and five non-target letters (H, K, M, W, or Z) arranged in random order (left). In the low cognitive load condition, the letter string consisted of six X’s or six N’s (right).

We recently examined the extent to which individual differences in self-regulatory capacity, indexed by resting HRV, were associated with the exertion of self-regulatory effort, indexed by phasic HRV, in response to emotional versus neutral distractors under different levels of cognitive load (Park et al., 2014). Participants were instructed to detect a target letter, either X or N, among letter strings superimposed on either fearful or neutral distractor faces. Letter strings consisted of six target letters under low load and one target letter and five non-target letters under high load (see Figure 4). People with higher and lower resting HRV were not different in task performance under low load. However, under high load, people with higher resting HRV were faster in conditions with neutral distractors, but not with fearful distractors, whereas people with lower resting HRV were slower in conditions with both fearful and neutral distractors (Park et al., 2013a). More importantly, people with lower resting HRV showed phasic HRV suppression, which suggested making an autonomic stress response, in the conditions with fearful distractor faces under both low and high load (Park et al., 2014). Thus, people with lower resting HRV appeared to make autonomic stress responses to trivial threat cues as if the cues were significant stressors. Conversely, people with higher resting HRV showed phasic HRV enhancement, which suggested making greater self-regulatory effort, under low load with fearful distractors and an absence of phasic HRV suppression under high load. Thus, people with higher resting HRV were capable of exerting emotion regulatory effort in response to fearful distractors under low load when processing resources were available under low load. However, people with higher resting HRV did not make an autonomic stress response, even under high load when task performance could be potentially stressful. The results are consistent with a previous study reporting that phasic HRV enhancement were observed in healthy individuals with higher resting HRV and phasic HRV suppression in patients suffering from social anxiety disorder characterized by lower resting HRV in response to emotionally negative stimuli (Gaebler et al., 2013). Our study further clarified that the availability of processing resources under different levels of load plays an important role in the effect of resting HRV on phasic HRV changes. As such, there is a growing body of literature suggesting that resting cardiac vagal tone is associated with the ability to flexibly control autonomic responses as well as selective attention, which may promote further emotion regulation and autonomic flexibility (Park et al., 2014).

## IMPLICATION OF HRV ON INDIVIDUAL WELL-BEING AND MENTAL HEALTH

Results of extensive research have indicated that low resting HRV is typically observed in people with various psychopathologies, including generalized anxiety disorder, panic disorder, depression, bipolar disorder, and schizophrenia (Thayer et al., 1996; Rottenberg et al., 2002; Friedman, 2007; Bär et al., 2008, 2009; Castro et al., 2008; Pittig et al., 2013). Furthermore, healthy individuals with lower resting HRV demonstrate heightened activity in the middle occipital gyrus and the cuneus during visual perception of emotional and neutral stimuli, which are typically observed in people with high risk of psychosis (Park et al., 2012a). Another line of research has indicated that cognitive processing of emotional stimuli may significantly contribute to the etiology and maintenance of various psychopathologies, such as anxiety, depression, and schizophrenia (Beauchaine et al., 2007). For example, it has been well established that people with high anxiety are characterized by attentional bias favoring threatening stimuli (Lee and Park, 2011). Schizophrenic patients also exhibit impaired visual perception of emotional facial expressions (see Edwards et al., 2002, for a review). However, there has been limited evidence linking HRV to bottom-up and top-down cognitive processing of emotional stimuli. Our research systematically examine the relationship between HRV and top-down and bottom-up visual perception and emotion attention and provide evidence that lower resting HRV is associated with hyper-vigilant and maladaptive bottom-up and impaired top-down cognitive response to emotional stimuli. These studies raise the possibility that lower resting HRV may be a predisposing factor that increases the susceptibility of developing emotional and psychiatric problems (Park et al., 2012a).

Furthermore, several studies provided evidence that resting HRV is associated with the ability to control autonomic responses (Butler et al., 2006; Segerstrom and Nes, 2007; Gaebler et al., 2013; Park et al., 2014). People with lower resting HRV showed an autonomic stress response, phasic HRV suppression, to fearful stimuli, indicating that they interpret seemingly mild stimuli as a significant stressor (Gaebler et al., 2013; Park et al., 2014). This hyper-vigilant autonomic stress response will trigger a cascade of psychological and physiological defensive responses, which eventually puts wear and tear on a host of physiological systems (Park et al., 2014). Indeed, lower resting HRV has been frequently observed in people suffering from various health problems, such as hypertension, diabetes, high cholesterol, obesity, arthritis, and some cancers (Thayer et al., 1996; Friedman and Thayer, 1998; Park et al., 2014). Therefore, the failure in effective cognitive processing of emotion stimuli may impact not only mental and psychology well-being but also physical health.

## CONCLUSION

The current review presented evidence of an underlying interaction between individual differences in cardiac vagal tone and top-down and bottom-up cognitive processing of emotional stimuli, which promotes further regulatory behaviors and autonomic flexibility. To further dissociate top-down and bottom-up mechanisms, we utilized stimuli with different spatial frequency ranges designed to tap into either top-down or bottom-up neural mechanisms of emotional processing. These studies provide evidence that higher resting HRV is associated with flexible and adaptive top-down and bottom-up cognitive processing, which facilitates effective emotion regulation. In contrast, lower resting HRV is associated with hyper-vigilant and maladaptive bottom-up and impaired top-down cognitive response to emotional stimuli, which is detrimental to emotion regulation. The results of these studies raise the possibility that maladaptive cognitive processing of emotional stimuli observed in people with lower HRV may be detrimental to emotional and physical health, which explains why people with a wide range of psychopathologies and health issues exhibit lower HRV.

## Conflict of Interest Statement

The authors declare that the research was conducted in the absence of any commercial or financial relationships that could be construed as a potential conflict of interest.
